# From Natural Methylation to Versatile Alkylations Using Halide Methyltransferases

**DOI:** 10.1002/cbic.202100153

**Published:** 2021-05-10

**Authors:** Qingyun Tang, Ioannis V. Pavlidis, Christoffel P. S. Badenhorst, Uwe T. Bornscheuer

**Affiliations:** ^1^ Institute of Biochemistry University of Greifswald Felix-Hausdorff-Str. 4 17489 Greifswald Germany; ^2^ Dept. of Chemistry University of Crete Voutes University Campus 70013 Heraklion Greece

**Keywords:** alkylation, alkyl iodide, halide methyltransferase, methylation, SAM analogue

## Abstract

Halide methyltransferases (HMTs) enable the enzymatic synthesis of *S*‐adenosyl‐l‐methionine (SAM) from *S*‐adenosyl‐l‐homocysteine (SAH) and methyl iodide. Characterisation of a range of naturally occurring HMTs and subsequent protein engineering led to HMT variants capable of synthesising ethyl, propyl, and allyl analogues of SAM. Notably, HMTs do not depend on chemical synthesis of methionine analogues, as required by methionine adenosyltransferases (MATs). However, at the moment MATs have a much broader substrate scope than the HMTs. Herein we provide an overview of the discovery and engineering of promiscuous HMTs and how these strategies will pave the way towards a toolbox of HMT variants for versatile chemo‐ and regioselective biocatalytic alkylations.

## Graphical abstract

**Naturally occurring** and engineered promiscuous halide methyltransferases (HMTs) enable *S*‐adenosyl‐l‐methionine analogues to be enzymatically synthesised and recycled, using simple and readily available alkyl iodides as alkyl donors. In combination with promiscuous methyltransferases (MTs), they dramatically expand the bioalkylation toolbox.

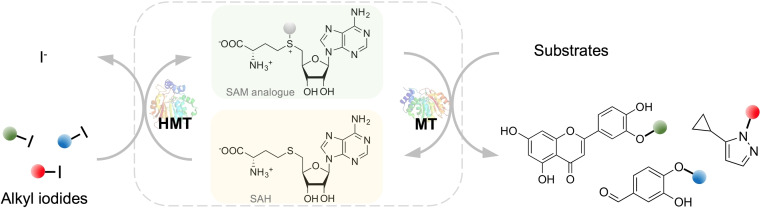

## Background

**Scheme 1:**
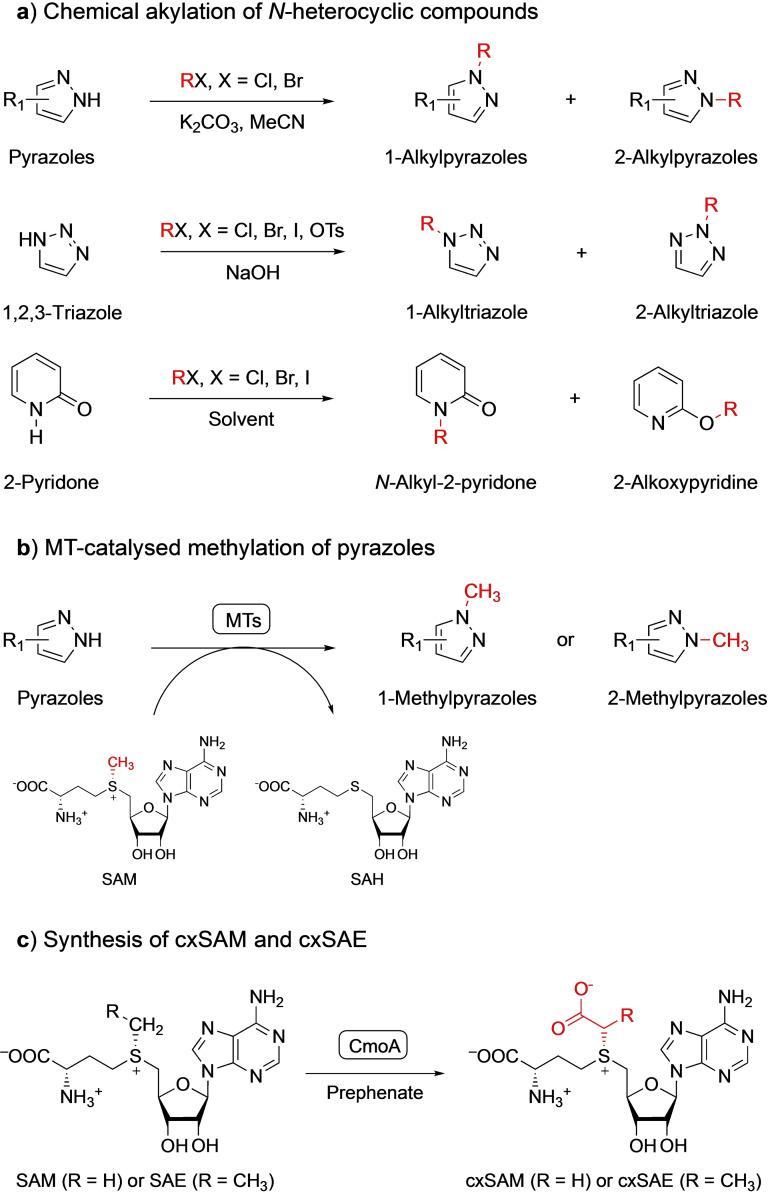
a) Due to tautomerisation of heteroatoms, chemical alkylation of *N*‐heterocyclic compounds like pyrazoles, triazole, and pyridone usually leads to mixtures of alkylated products. b) Regioselective methylation of pyrazoles catalysed by SAM‐dependent MTs. By using different MT variants, either 1‐methylpyrazoles or 2‐methylpyrazoles can be synthesised with regioisomeric ratios up to 99 %. c) CxSAM is a rare example of a naturally occurring SAM analogue. CxSAM is synthesised from prephenate and SAM by CmoA. Starting from *S*‐adenosyl‐l‐ethionine (SAE), the cxSAE analogue can be synthesised.

The “magic methyl” effect refers to the ability of well‐placed methyl groups to dramatically alter the physical and biological properties of a molecule.^[1]^ This makes methylation one of the most atom‐efficient strategies for modulation of biological effects.[^1a^, ^2^] Going beyond methylation, structural diversification of natural products by alkyl‐diversification would create libraries of compounds with profoundly enhanced properties. However, catalyst‐controlled chemo‐ and regioselective alkylation of molecules bearing two or more heteroatoms with similar reactivities is still a major challenge in organic synthesis. This is particularly true for *N*‐heterocyclic compounds like pyrazoles, triazoles, and pyridones, since tautomerisation can lead to different heteroatoms (e. g. nitrogen and oxygen) having similar reactivities (Scheme 1a).^[3]^ Since drug molecules often contain amines, *N*‐heterocycles, and unprotected polar groups, selective heteroatom alkylation is a reaction on many organic chemists′ wish list.^[4]^

Enzymes can address this challenge, due to their chemo‐, regio‐, and stereoselectivities.^[5]^ For example, the *S*‐adenosyl‐l‐methionine‐dependent methyltransferases (MTs) catalyse the highly chemo‐ and regioselective methylation of a broad range of biomolecules at C, O, N, S, and P atoms.^[6]^ Very recently, Hammer's group demonstrated the incredible potential of protein engineering by designing a panel of methyltransferases for the regioselective methylation of pyrazoles, with regioisomeric ratios up to 99 % (Scheme 1b).^[4b]^ Before, there were no chemical, or even biological, catalysts known capable of controlling pyrazole alkylation so precisely.^[4]^

**Scheme 2:**
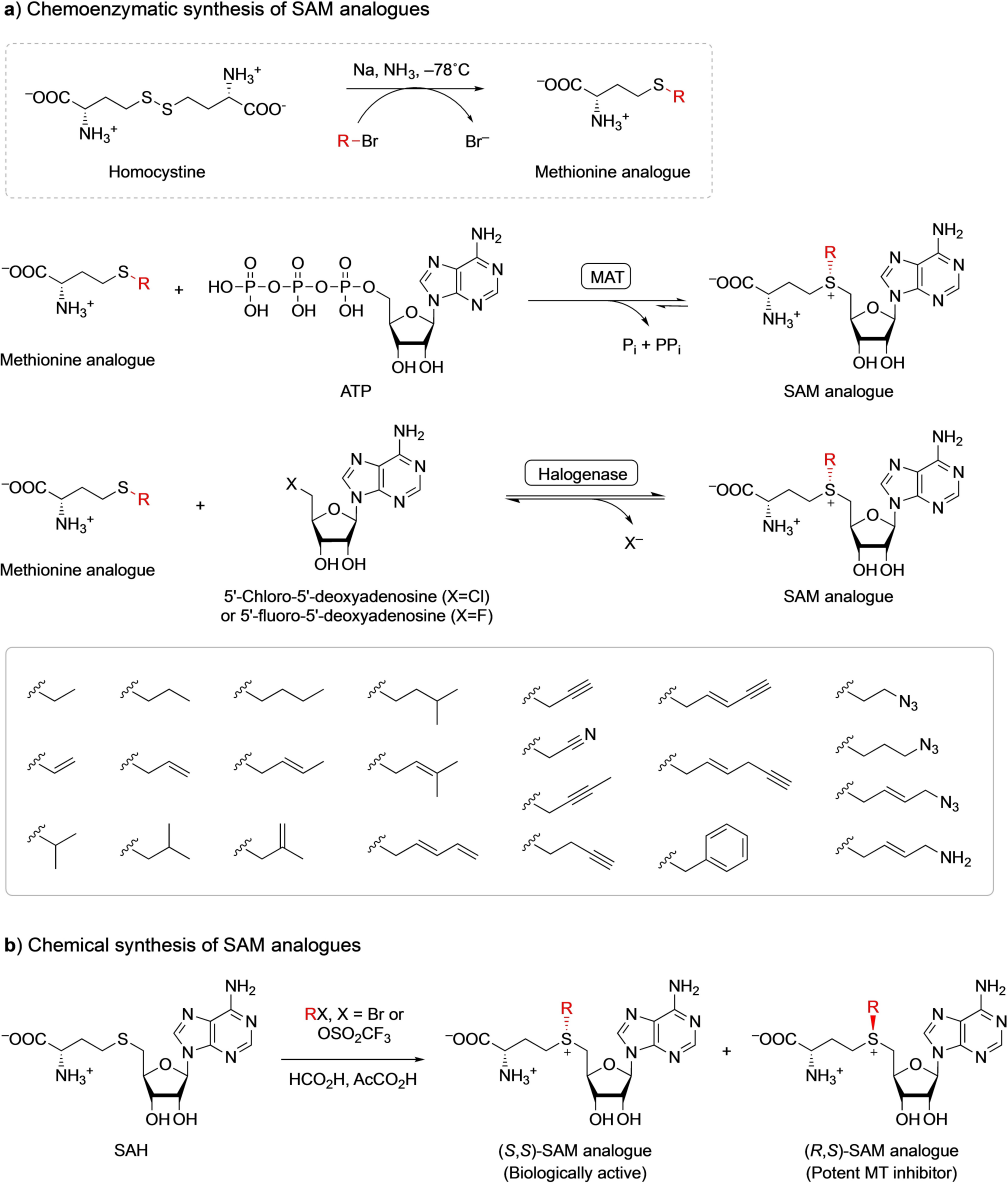
a) Chemoenzymatic synthesis of SAM analogues. Methionine analogues can be synthesized by reductive alkylation of homocystine. SAM analogues can be synthesized by MATs from methionine analogues and ATP, or by halogenases from methionine analogues and 5′‐chloro‐5′‐deoxyadenosine or 5′–fluoro‐5′‐deoxyadenosine. The box shows alkyl groups of SAM analogues produced by MATs and halogenases. b) Chemical synthesis of SAM analogues. SAH can be directly alkylated using alkyl triflates or alkyl bromides, resulting in a mixture of epimers.

Interestingly, like many naturally‐occurring MTs, Hammer's biocatalysts are promiscuous, in terms of the alkyl donor, and can transfer a range of alkyl groups, as long as the corresponding *S*‐adenosyl‐l‐methionine (SAM) analogues are available.[^6c^, ^7^] Unfortunately, SAM analogues are rare in nature and hard to synthesise. A prominent exception is carboxy‐SAM (cxSAM, Scheme 1c), which was identified as a co‐purified ligand in the crystal structure of CmoA, the enzyme that synthesises cxSAM from SAM and prephenate.^[8]^ CxSAM is used by the carboxymethyl transferase CmoB for tRNA alkylation.^[9]^ A carboxy‐*S*‐adenosyl‐l‐ethionine (cxSAE) analogue (Scheme 1c) has been synthesised from ethionine, using a human methionine adenosyltransferase (MAT) and CmoA.^[8]^ The MAT uses ethionine, a methionine analogue, to produce SAE, the corresponding SAM analogue. MATs use ATP as source of the adenosyl moiety and are remarkably promiscuous with regards to methionine analogues, allowing a broad range of SAM analogues to be synthesised (Scheme 2a).^[10]^ Halogenases have also been used to synthesise SAM analogues from methionine analogues. These enzymes use 5′‐chloro‐5′‐deoxyadenosine or 5′‐fluoro‐5′‐deoxyadenosine as donors of the adenosyl moiety (Scheme 2a).^[11]^ MATs and halogenases are often coupled with MTs in one‐pot cascade reactions to form alkylated products. Details about these enzymes and their applications in biocatalytic alkylation cascades have been described in recent reviews.[^6c^, ^7e^, ^12^] Methionine analogues need to be chemically synthesised as they are not readily available (Scheme 2a).[^10a^, ^11c^, ^13^] The harsh chemical synthesis conditions are not compatible with biotransformations and therefore have to be performed separately, limiting their application. SAH can be directly alkylated using alkyl triflates or alkyl bromides, but this results in a mixture of (*S*,*S*)‐ and (*R*,*S*)‐epimers, the latter being potent MT inhibitors (Scheme 2b).^[14]^ Furthermore, residual SAH is also a potent MT inhibitor.^[15]^ Consequently, chemically synthesised SAM analogues have to be purified by – non‐trivial – chromatography, limiting the application of this approach.[^7a^, ^16^]

## Regeneration of SAM Analogues

SAM has to be used in stoichiometric amounts, producing an equivalent amount of SAH, which is a potent MT inhibitor.^[15]^ A fundamental problem is that SAM‐dependent enzymes transfer a 15 Da methyl group using a 384 Da handle that has to be degraded because it inhibits MT activity.^[17]^ Poor atom economy has therefore always been a significant drawback of SAM‐dependent methylations. A landmark publication by the Andexer group showed that SAM could be regenerated using a five‐enzyme cascade, starting with the hydrolysis of SAH to adenosine and homocysteine, followed by three phosphorylations of adenosine to form ATP, which is used by an MAT to convert methionine to SAM. Unfortunately, this cascade fails after 11 cycles, produces homocysteine as waste, and depends on methionine and its analogues as alkyl donors.^[18]^

**Scheme 3:**
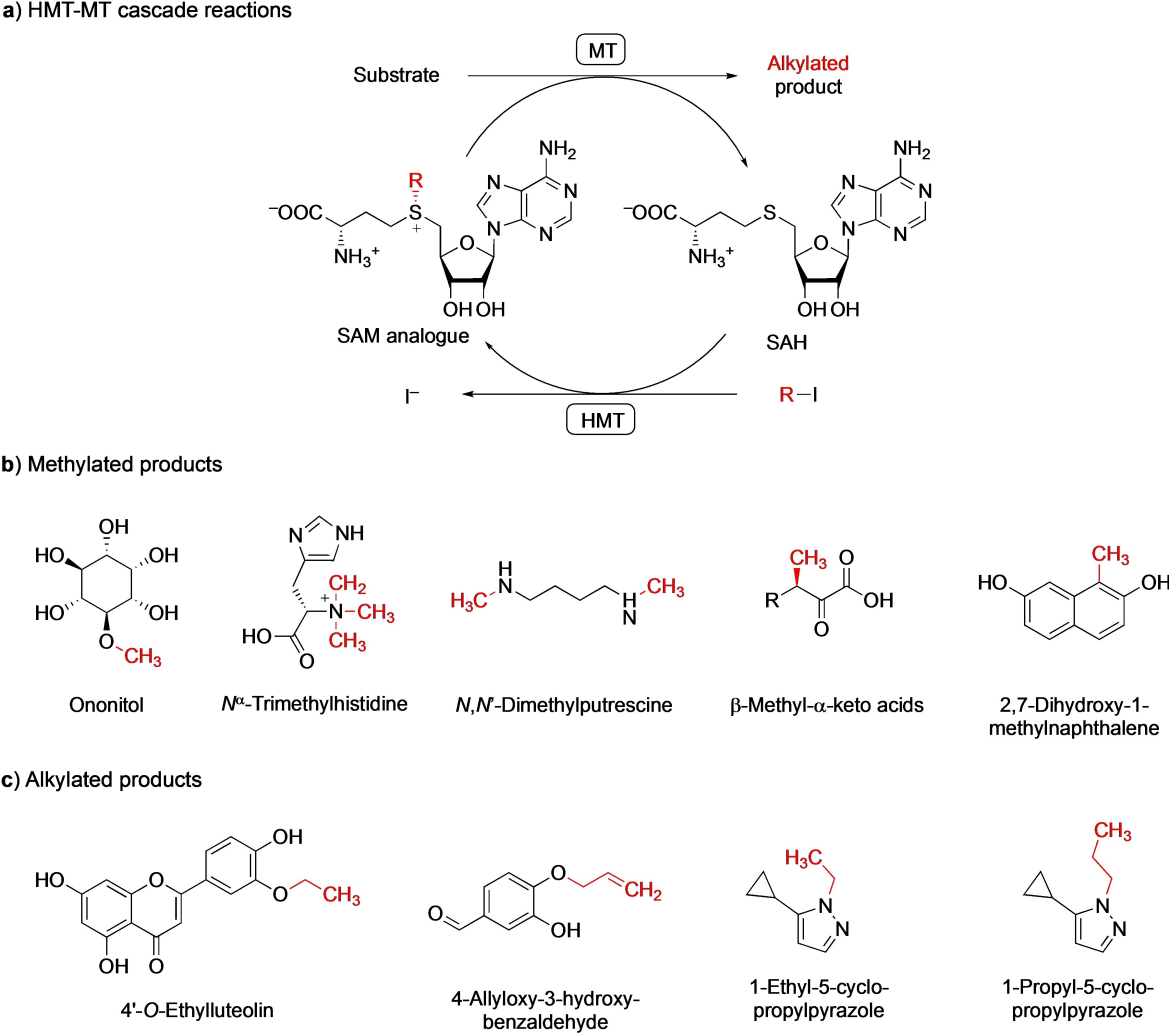
a) HMT‐MT cascade reactions produce iodide as the only by‐product, making these processes atom‐efficient. b) Methylated products[^17^, ^20^] and c) alkylated products[^4b^, ^21^] obtained by HMT‐MT cascade reactions. The introduced methyl or alkyl groups are coloured red.

Recently, Liao and Seebeck described a one‐enzyme system for SAM recycling (Scheme 3a).^[17]^ Halide methyltransferases (HMTs) use SAM to methylate halide ions, annually releasing megatons of methyl halides into the atmosphere.^[19]^ Liao and Seebeck realized that methyl halide synthesis is driven by the evaporation of volatile methyl halides and the metabolic degradation of SAH. Therefore, they could employ the reaction in reverse, which is thermodynamically favoured, enabling the production of SAM from SAH and methyl iodide, with iodide as the sole by‐product (Scheme 3a). A stoichiometric amount of methyl iodide is provided as methyl group donor and a catalytic amount of SAH is recycled up to 580 times.^[17]^ The HMT‐synthesized SAM was utilized by different *O*‐, *N*‐ and *C‐*MTs to produce several regioselectively methylated products (Scheme 3b).[^17^, ^20^]

## Promiscuous HMTs

Recently, the Bornscheuer^[21]^ and Hammer^[4b]^ groups demonstrated that HMTs can also be used for the synthesis and regeneration of a range of SAM analogues. These HMTs, combined with alkyl‐promiscuous MTs, produced the regioselectively alkylated products 4′‐*O*‐ethylluteolin, 4‐allyloxy‐3‐hydroxybenzaldehyde, 1‐ethyl‐5‐cyclopropylpyrazole, and 1–propyl‐5‐cyclopropylpyrazole (Scheme 3c). In our work, more than 40 regeneration cycles were achieved for the preparative scale alkylation of flavonoid and phenolic compounds, using 100 μM of SAH.^[21]^ Importantly, these cycle numbers may well be underestimated, as additional SAH added to the reactions may not have been necessary. The traces of SAH and/or SAM bound to purified proteins are sufficient for bioalkylation cascades.^[4b]^

A key realisation was that, just like methyltransferases in general, HMTs should be alkyl‐promiscuous, enabling them to accept donors other than methyl iodide. While the *Chloracidobacterium thermophilum* HMT used by Liao and Seebeck did not have significant ethyltransferase activity, the enzymes from *Arabidopsis thaliana*, *Raphanus sativus*, *Aspergillus clavatus*, *Batis maritima*, and *Synechococcus elongatus* did.[^4b^, ^21^] As we predicted, even more promiscuous HMTs can be found in nature, and Bengel et al. reported that the *Aspergillus clavatus* HMT had significant propyltransferase activity and could even produce a small amount of the butyl analogue of SAM (Table 1).^[4b]^ This enzyme was identified by screening only seven of the HMTs described in the literature, suggesting that even more useful HMTs could be found in nature. Therefore, we suggest further investigation of the 89 HMTs that have already been functionally characterised in the past.^[22]^ However, it is very unlikely that an HMT would be so promiscuous that it could be used for any alkyl group of interest. Therefore, HMT engineering will probably play an important role in the future of bioalkylation.

**Table 1:** Conversion [%] of SAH and alkyl iodides to SAM analogues by promiscuous HMTs.

Enzyme^[a]^	Methyl iodide	Ethyl iodide	Ethyl bromide	Propyl iodide	Allyl iodide	Butyl iodide
*Arabidopsis thaliana* HMT (WT)^[b]^	100	44	1	0	–	0
*Arabidopsis thaliana* HMT (V140T)^[c]^	–	90	–	50	71	–
*Aspergillus clavatus* HMT^[b]^	100	78	99	31	–	3
*Batis maritima* HMT^[b]^	99	81	100	1	–	0
*Synechococcus elongatus* HMT^[b]^	100	61	21	3	–	1

[a] The *Raphanus sativus* HMT was also tested by Tang et al.^[21]^ but is not mentioned because activity was only determined in U/mg and conversion similar to other data in this table was not determined. [b] Reactions were performed with crude *E. coli* cell lysate, 1 mM SAH, and 40–80 equivalents of alkyl halides at 25 °C for 20 h, as described by Bengel et al.^[4b]^ [c] Reactions were performed on preparative scale with purified enzyme, 15 mg SAH and 8 equivalents of alkyl iodides at 25 °C for 24 h, as described by Tang et al.^[21]^

## HMT Engineering

Utilizing a very sensitive iodide assay, we identified a variant (V140T) of the *Arabidopsis thaliana* HMT that can synthesise the ethyl, propyl, and allyl analogues of SAM (Table 1).^[21]^ We used ethyl iodide as substrate for screening our site‐directed mutagenesis libraries, as the wild‐type *Arabidopsis thaliana* HMT already had activity towards ethyl iodide and therefore the chances of finding improved variants were good. We believe that screening larger libraries using bulkier substrates like butyl‐ or benzyl iodide would lead to the identification of variants capable of efficiently producing the corresponding SAM analogues. The results we obtained by screening with ethyl iodide led to two important conclusions. First, screening using a slightly larger donor (e. g., ethyl vs. methyl iodide) can result in the identification of variants active on even larger substrates (e. g., propyl and allyl iodide). Second, the V140T variant was generally the best catalyst for transferring all the alkyl groups we tested. This suggests that a single substitution can make an HMT promiscuous and that engineering robust general alkyl transfer catalysts should be possible.

We originally tested the HMTs from *Chloracidobacterium thermophilum* (the enzyme used by Liao and Seebeck), *Raphanus sativus* (highest activity towards methyl iodide), and *Arabidopsis thaliana*.[^17^, ^23^] The *Arabidopsis thaliana* HMT was engineered because it had the highest activity towards ethyl iodide between the three candidates and because its 3D structure was available, which facilitated semi‐rational protein engineering. Currently, this is still the only solved HMT structure.^[24]^ The next most similar structure in the PDB is that of murine thiopurine *S*‐methyltransferase, with only 20 % sequence identity.^[25]^ Further HMT engineering would benefit from detailed structural and mechanistic understanding, suggesting that solving more HMT structures will be important.

**Figure 1:**
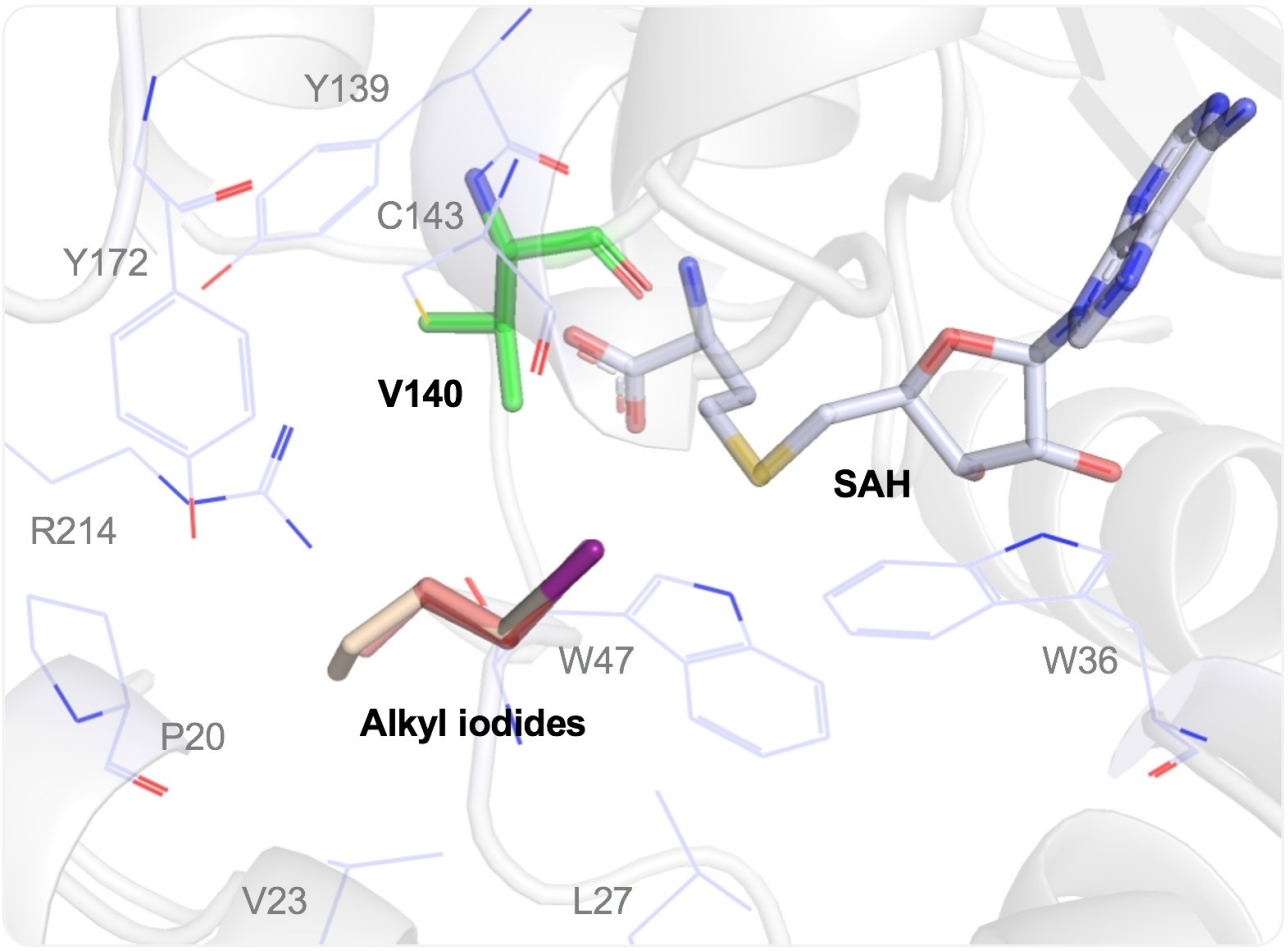
Crystal structure of *Arabidopsis thaliana* HMT with the ligand SAH and docked methyl, ethyl, propyl, and butyl iodide in the active site. V140, SAH, and alkyl iodides are shown as sticks. Other sites mutated by Tang et al.^[21]^ are shown as lines.

While molecular docking suggested that the *Arabidopsis thaliana* HMT can accommodate even butyl iodide in its spacious active site, substrates larger than ethyl iodide are not converted (Figure 1).^[21]^ Currently, it is believed that SAM‐dependent MTs catalyse alkyl transfer by binding the substrates (SAM analogue and acceptor) in reactive conformations, pre‐aligning electron orbitals for efficient S_N_2 displacement.^[26]^ This is likely also the case for HMTs, but we are not aware of detailed mechanistic studies. The lower activity with larger alkyl halides probably results from less efficient binding of the larger substrates in the same reactive conformation as methyl iodide.

A better understanding of the natural functions of HMTs would also facilitate application and engineering of these enzymes. Suggestions that the function of HMTs is to regulate intracellular halide concentrations seem highly unlikely; some studies have suggested that the formation of methyl halides is a ′metabolic accident′.^[27]^ Generally, HMT activities depend on substrate in the order SCN^−^, HS^−^, I^−^>Br^−^>Cl^−^, which corresponds to the nucleophilicity of the anions.[^19b^, ^19c^, ^22^, ^23^, ^28^]

A few studies have suggested that the intended substrates of HMTs are toxic thiols resulting from the hydrolysis of, for example, glucosinolates.^[28a]^ The catalytic efficiencies of *Arabidopsis thaliana* HMT against SCN^−^ and (NH_4_)_2_S are even five orders of magnitude higher than against Cl^−^, which indicates that methylation of halides might be an unspecific side‐reaction.[^27^, ^28b^] This is a significant consideration because it suggests that halide‐methyl transfer is a broadly distributed promiscuous activity of methyltransferases and testing a much larger number of MTs for promiscuous HMT activity is advisable. HMT and thiol methyltransferase (TMT) protein sequences share similarities and phylogenetic analysis suggests that they are evolutionarily related.[^24^, ^28b^] Both HMTs (E.C. 2.1.1.165) and TMTs (E.C. 2.1.1.9) can methylate halide ions, bisulfide (HS^−^), and thiocyanate (SCN^−^).[^19b^, ^19c^, ^23a^, ^28^] Therefore, TMTs should also be investigated for conversion of alkyl iodides to SAM analogues.

## Future Directions

**Scheme 4:**
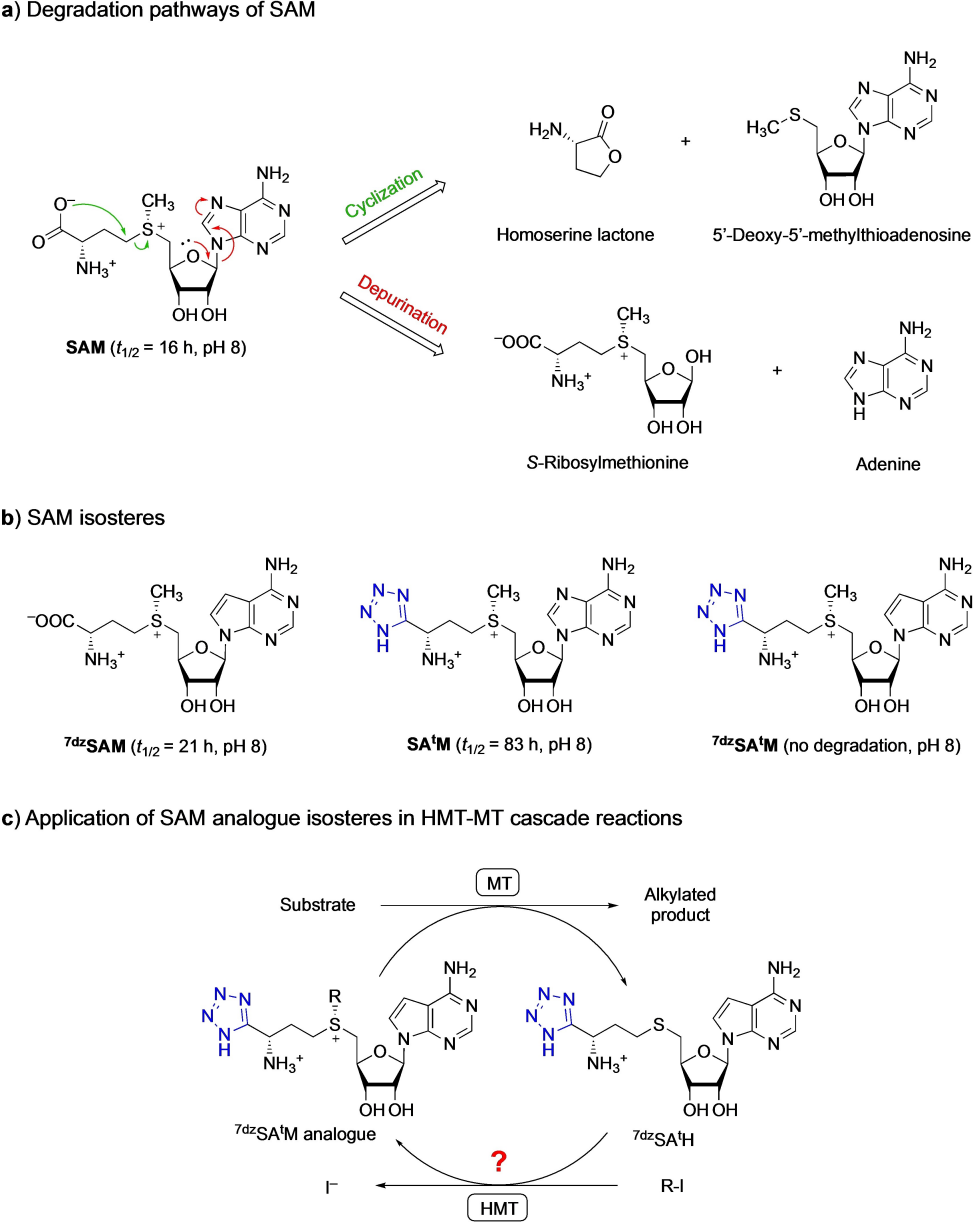
a) The two main degradation pathways of SAM via intramolecular cyclization (green) and depurination (red). b) Structures of the SAM isosteres SA^t^M, with a tetrazole replacing the carboxyl group (coloured blue), S^7dz^AM, with 7‐deazaadenosine replacing the adenosine moiety of SAM, and S^7dz^A^t^M, combining these modifications. c) An important question for future research is whether HMTs can alkylate these stable SAM isosteres in HMT‐MT cascade reactions to achieve more regeneration cycles.

The inherent chemical instability of SAM under physiological conditions (half‐life of about 16 h at pH 8) is a frequently discussed concern, complicating for example the purification of SAM analogues.^[29]^ Intramolecular cyclization results in the formation of 5’‐deoxy‐5’‐methylthioadenosine and homoserine lactone, while depurination results in degradation to adenine and *S*‐ribosylmethionine (Scheme 4a).^[30]^ Now that efficient systems for SAM analogue regeneration from SAH are available,[^4b^, ^17^, ^21^] stability becomes a more significant consideration than the degradation of the coproduct SAH to avoid inhibition of methyltransferases. Huber et al. used an MAT to produce the more stable SAM isosteres S^7dz^AM (half‐life of 21 h), SA^t^M (half‐life of 83 h), and S^7dz^A^t^M (no degradation at pH 8) (Scheme 4b).^[31]^ These isosteres could be used by a prototypical Class I model MT^[31]^ but it is currently not known whether HMTs can accept these isosteres in HMT‐MT cascade reactions to achieve more regeneration cycles (Scheme 4c). Furthermore, it is exciting to think about applying “bump and hole” strategies for making SAH analogues that are selectively activated by mutant HMTs and used exclusively by mutant MTs, thus creating ideal bioorthogonal alkylation cascades.[^7c^, ^32^]

It remains to be seen how tolerant HMTs are to modifications of the SAH moiety. For bioorthogonal alkylation cascades, an important question is whether haloalkanes are compatible with *in vivo* cascades. What we know from the development of an *in vivo* selection for dehalogenase activity is that *E. coli* can replicate in the presence of millimolar concentrations of alkyl halides.^[33]^ Bayer et al. demonstrated that *Saccharomyces cerevisiae* is resistant to 5 g/L of methyl iodide. Diffusion of these hydrophobic compounds into cells should allow SAM analogue production by intracellular HMTs.

Chemical methylation agents like methyl iodide are intrinsically toxic, so the demand for benign and environmentally friendly alkylation methods still exists. Fortunately, the toxicity, electrophilicity, and volatility of alkyl iodides decrease with longer alkyl chains. HMT activity on less electrophilic alkyl donors like alkyl bromides and chlorides would also enable more selective alkylation. Interestingly, ethyl bromide has been shown to be well accepted as alkyl donor by the *Aspergillus clavatus*, *Synechococcus elongatus*, and *Batis maritima* HMTs (Table 1).^[4b]^ It would be very interesting to see whether HMTs can be engineered for higher activity towards these substrates, and our previously described ultrasensitive bromide assay would facilitate such engineering.^[34]^

Another exciting avenue for further research is *in situ* generation of alkyl halides, decreasing the dependence on alkyl iodides. An emerging possibility is the use of non‐haem iron halogenases for the stereo‐ and regioselective halogenation of non‐activated sp^3^‐carbon atoms.^[35]^ A potential limitation is the preference of these enzymes for chlorination. A simple general approach bridging the chloride‐forming halogenases with the iodide‐preferring HMTs would be enzymatic transhalogenation, which uses “transhalogenases” to convert alkyl chlorides to alkyl iodides.^[36]^ In this light, biocatalytic alkylation is expected to move beyond mere alkyl‐diversification towards a generalised ability to selectively link two molecular scaffolds.

## Conclusions

The recently described promiscuous halide MTs have expanded the bioalkylation toolkit and enable selective ethylation, propylation, and allylation of target compounds. Furthermore, our group has demonstrated that the substrate scope of HMTs can be expanded, and we believe that a combination of high‐throughput screening, facilitated by our halide assays,[^21^, ^34^] with rational library design, using computational tools like FuncLib,[^4b^, ^37^] will rapidly expand the range of easily accessible SAM analogues. However, detailed mechanistic understanding of how HMTs function is currently lacking. Determining the crystal structures and catalytic mechanism of more HMTs would enable more rational design, perhaps allowing SAM analogues of currently unimaginable complexity to be enzymatically synthesised from alkyl halides. We believe that HMTs might soon displace MATs from their prominent position in biocatalytic alkylation cascades.

## Conflict of interest

The authors declare no conflict of interest.
